# Fabrication of Carbon Nanofiber Incorporated with CuWO_4_ for Sensitive Electrochemical Detection of 4-Nitrotoluene in Water Samples

**DOI:** 10.3390/s23125668

**Published:** 2023-06-17

**Authors:** Ganesh Abinaya Meenakshi, Subramanian Sakthinathan, Te-Wei Chiu

**Affiliations:** 1 Department of Materials and Mineral Resources Engineering, National Taipei University of Technology, No. 1, Section 3, Chung-Hsiao East Road, Taipei 106, Taiwan; abimeenakshi1998@gmail.com (G.A.M.); sakthinathan1988@gmail.com (S.S.); 2 Institute of Materials Science and Engineering, National Taipei University of Technology, No. 1, Section 3, Chung-Hsiao East Road, Taipei 106, Taiwan

**Keywords:** CuWO_4_, CNF, electrochemical sensor, 4-nitrotoluene, real sample analysis

## Abstract

In the current work, copper tungsten oxide (CuWO_4_) nanoparticles are incorporated with carbon nanofiber (CNF) to form CNF/CuWO_4_ nanocomposite through a facile hydrothermal method. The prepared CNF/CuWO_4_ composite was applied to the electrochemical detection of hazardous organic pollutants of 4-nitrotoluene (4-NT). The well-defined CNF/CuWO_4_ nanocomposite is used as a modifier of glassy carbon electrode (GCE) to form CuWO_4_/CNF/GCE electrode for the detection of 4-NT. The physicochemical properties of CNF, CuWO_4_, and CNF/CuWO_4_ nanocomposite were examined by various characterization techniques, such as X-ray diffraction studies, field emission scanning electron microscopy, EDX-energy dispersive X-ray microanalysis, and high-resolution transmission electron microscopy. The electrochemical detection of 4-NT was evaluated using cyclic voltammetry (CV) the differential pulse voltammetry detection technique (DPV). The aforementioned CNF, CuWO_4_, and CNF/CuWO_4_ materials have better crystallinity with porous nature. The prepared CNF/CuWO_4_ nanocomposite has better electrocatalytic ability compared to other materials such as CNF, and CuWO_4_. The CuWO_4_/CNF/GCE electrode exhibited remarkable sensitivity of 7.258 μA μM^−1^ cm^−2^, a low limit of detection of 86.16 nM, and a long linear range of 0.2–100 μM. The CuWO_4_/CNF/GCE electrode exhibited distinguished selectivity, acceptable stability of about 90%, and well reproducibility. Meanwhile, the GCE/CNF/CuWO_4_ electrode has been applied to real sample analysis with better recovery results of 91.51 to 97.10%.

## 1. Introduction

4-Nitrotoluene (4-NT), also known as para nitrotoluene, is one of the noticeable intermediates of nitro-aromatic compounds (NAC). It has been widely used in the production of dyes, medicaments, synthetic fibers, pesticides, paints, rubber, and explosives [1]. In addition, 4-NT has been used as an intermediate in the synthesis of chemicals, such stilbene, p-nitrobenzoic acid, p-nitro benzaldehyde, and p-toluidine, which are consumed as starting materials for agricultural, drug, and dye industries. Owing to the extensive industrial application, 4-NT discharged into water and soil lead to contamination of soil and water causing hazardous effects on the ecological system either directly or indirectly [2]. Further, the prolonged consumption of 4-NT leads to severe health issues such as cardiovascular troubles, emphysema, dizziness, skin irritation, intoxication, endocrine disruption, and distraction in the central nervous system. Thus, the detection of 4-NT has become a serious concern [3]. So far, numerous analytical techniques, such as chemiluminescence [4], gas chromatography [5], fluorescence spectroscopy [6], liquid chromatography [7], and electrochemical analysis, have been employed in the determination of 4-NT [2]. Among all, electrochemical analysis has various merits such as cost-effectiveness, online monitoring, accuracy, easy fabrication of electrodes, high sensitivity, and quick response [2].

Previous studies on electrochemical detection of 4-NT were engaged with carbon-based metal/metal oxide ion composite materials [8], molecularly imprinted polymers [9], metallic/mixed-metallic nanoparticles [10], and aptamer-based materials [11]. Nevertheless, most of these catalysts possess a limit of detection (LOD) at the micromolar level, electrode fouling, immoderate cost, and poor selectivity [12]. To overcome this issue, heterogeneous catalysts are considered a suitable material for electrochemical sensing [13]. In recent years, bimetallic tungstate is used in various fields as water splitting [14], photocatalysis [15], electrochemical sensors [16], hydrogen-evolution reactions [17], supercapacitors [18], and scintillators [19]. Different kinds of metal tungstate have been reported in the sensor application [20]. Metal tungstate has a superior electrical conductivity due to the occupation of the W atoms (10^−7^ to 10^−3^ S cm^−1^). CuWO_4_ is one of them, an n-type semiconductor with a small band gap of 2.2 eV and advantageous qualities such as being inexpensive, readily available, nontoxic, cost-effective electrodes for clean, well-renewable, and maintainable energy devices, as well as stable electrode material for electrocatalytic applications [21,22,23]. The water-splitting and stable photocatalytic properties of CuWO_4_ decorated with polypyrrole were reported by P. H. Dinh et al. [24]. CuWO_4_ has been used to modify an electrode material due to the wide surface area, high resistance, and high sensitivity, which opens up the possibility of investigating the potential of this material for use in electrochemical sensing applications. By utilizing the hybridization of carbon-based materials, the high-resistive characteristics of CuWO_4_ were lowered [25,26,27].

To improve the sensitivity and selectivity of the bare GCE electrode, metal oxides are combined with different carbonaceous materials, which can adequately promote charge transfer through a synergetic effect [28]. Generally, carbonaceous nanomaterials have a large specific surface area and a high surface–volume ratio. Moreover, these materials establish excellent biocompatibility, a massive rate of electron transfer, electrocatalytic properties, and interfacial adsorption features, which makes them distinguished from other materials in electrochemical sensing [29]. For instance, carbonaceous materials, viz., fullerenes, graphite, graphene, graphene oxide, carbon nano horns, carbon nanofibers (CNF), carbon nanotubes (CNT), and carbon quantum dots, were reported [30]. For electrochemical sensing applications, the combination of bimetallic tungstate with carbon-based materials, such as graphene oxide (GO), carbon nanofibers (CNFs), carbon nanotubes (CNTs), ordered mesoporous carbon (OMC), and graphitic carbon nitride (*g*-C_3_N_4_), is becoming more and more common [31,32,33].

In recent times, carbon nanofiber has emerged as an ideal material because of its properties, such as good electrical and thermal conductivity [34,35]. Moreover, the CNF can easily bond with high-conductive metal materials to generate composite materials, which are often used as an electrocatalyst for electrochemical sensing [36]. Jeyaraman et al. have reported the CuWO_4_/Sg-C_3_N_4_ by ultrasonication method for the electrochemical detection of nitrofurazone [37]. Pavithra et al., have investigated the electrochemical sensing and photocatalysis of nitrofurazone by the ZnWO_4_-CuWO_4_ composite [38]. The electrochemical detection of nicotine using the composite CuWO_4_/rGO was demonstrated by A. Karthiga et al. [39]. CuWO_4_@MoS_2_ and chitosan-Au nanoparticles were proposed to detect cortisol by C. Nong et al. [40]. Subsequently, it is essential to fabricate a nanocomposite with remodeled electrodes together with a preferable surface area, sensitivity, reproducibility, and more notable electrocatalytic materials for the detection of 4-NT.

To achieve the aforementioned properties, GCE is modified with CNF/CuWO_4_ nanocomposites made to act as an electrochemical sensor for the detection of 4-NT. Herein, we reported the preparation of a CuWO_4_ composite by a facile, novel, and inexpensive hydrothermal method. On the other hand, CuWO_4_/CNF nanocomposites were prepared by a simple ultrasonication method which is less time-consuming, more convenient, free of solvent and a more productive method. To my best of knowledge, CuWO_4_/CNF nanocomposites have not reported yet towards the detection of 4-NT. The prepared CNF, CuWO_4,_ and CNF/CuWO_4_ nanocomposite have been studied by various analytical techniques. The prepared CuWO_4_/CNF/GCE electrode successfully detects 4-NT at a low LOD with high sensitivity, selectivity, repeatability, and reproducibility. The CuWO_4_/CNF/GCE electrode was tested in water samples for exhibiting good recovery results.

## 2. Materials and Methods

All the chemicals used for this experiment were analytical grade without further purification. The chemicals Copper (II) Nitrate Hexahydrate ((Cu (NO_3_)_3_·6H_2_O) and Sodium tungstate dihydrate (Na_2_WO_4_·2H_2_O) were obtained from Sigma Aldrich Chemical Company, Taiwan. The PBS solution was prepared by NaH_2_PO_4_ and Na_2_HPO_4_. Different characterization studies were used to explore the prepared materials. The pH level was monitored by using a Suntex pH meter at room temperature (SP-2100). Voltammetry experiments were conducted using a three-electrode system, with an Ag/AgCl electrode acting as a reference electrode, GCE as a working electrode (surface area = 0.071 cm^2^), and a platinum wire acting as an auxiliary electrode. A scanning electron microscope was used for surface morphology analysis, and energy-dispersive X-ray spectroscopy was used for the elemental analyses (FESEM-EDX, JEOL JSM-7610F, Tokyo, Japan, and Hitachi Regulus 8100), X-ray diffraction tests (XRD, D2 Phaser, Bruker, Billerica, MA, USA, λ = 1.540 Å) were used for the phase structure. The CHI 1211B (CH Instruments Co., Austin, TX, USA) electrochemical workstation with cyclic voltammetry (CV), differential pulse voltammetry (DPV), and amperometry technique (i–t) was used for electrochemical studies.

### 2.1. Preparation of CuWO_4_/CNF Nanocomposite

Firstly, 1M of Cu (NO_3_)_2_ was dissolved in 20 mL of DI water and subjected to thorough stirring. Simultaneously, 1M of Na_2_WO_4_ solution was prepared using the aforementioned procedure. Then, the prepared Na_2_WO_4_ solution was added dropwise to the Cu (NO_3_)_2_ solution and kept for homogeneous stirring to form the CuWO_4_ solution. To improve the status of the reaction, the homogeneous CuWO_4_ solution is transferred to a Teflon-lined, stainless-steel autoclave and placed in the hydrothermal oven at 120 °C for 5 h. After the hydrothermal reaction, the hot autoclave was allowed to cool down to room temperature, and the collected solution was washed with ethanol and DI water several times to obtain the precipitate. Then, the precipitate was dried at 60 °C for 24 h to obtain CuWO_4_ nanoparticles. Secondly, the synthesized CuWO_4_ nanoparticles were incorporated with carbon nanofibers through the facile ultra-sonication method to make up CuWO_4_/CNF nanocomposite.

### 2.2. Fabrication of GCE/CNF/CuWO_4_ Electrode

The following methodology was adopted to modify the CuWO_4_/CNF/GCE electrode. CuWO_4_ nanoparticles (1 mg) and CNF (0.25 mg) were dispersed evenly in DMF solution in the ratio of 4:1 and sonicated for 1 h to generate CuWO_4_/CNF nanocomposite via noncovalent interaction. On the other hand, the GCE is polished with an alumina slurry well for 15 min and washed with ethanol through ultra-sonication to obtain a cleaned GCE surface. Following these steps, about 6 μM is drop cast onto the precleaned GCE surface and dried at room temperature to acquire CuWO_4_/CNF/GCE electrode. The fabricated electrode is imposed for further electrochemical studies of 4-NT detection.

## 3. Results

### 3.1. XRD Studies

**Figure 1:**
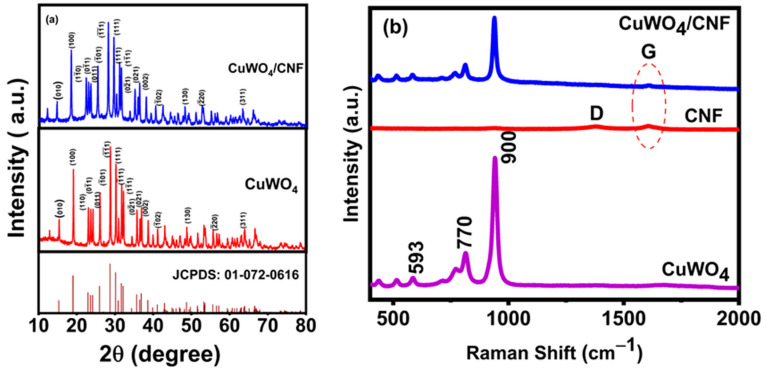
(**a**) XRD patterns of CuWO_4_ and CNF/CuWO_4_ nanocomposite. (**b**) Raman spectra of CuWO_4_, CNF, and CNF/CuWO_4_ nanocomposite.

The XRD analysis was applied to obtain the crystallinity of the prepared material, which is portrayed in Figure 1. Crystallinity and phase purity of CuWO_4_ and CuWO_4_/CNF were compared. The CuWO_4_ exhibited related strong diffraction peaks were attributed at 2θ values of 15.28°, 18.92°, 19.02°, 22.8°, 23.3°, 24.13°, 26.01, 28.63°, 30.15°, 30.91°, 31.67°, 32.0°, 34.42°, 35.61°, 36.48°, 36.48°, 36.92°, 38.55°, 39.86°, 41.16°, 42.93°, 48.70°, 51.55°, 53.29°, and 53.54°, corresponding to (010), (001), (100), (110), (0$\overset{-}{1}$1), (011), ($\overset{-}{1}$01), ($\overset{-}{1}$$\overset{-}{1}$1), (111), (020), ($\overset{-}{1}$11), (1$\overset{-}{1}$1), (120), (021), (021), (002), ($\overset{-}{1}$20), (210), ($\overset{-}{1}$02), ($\overset{-}{1}$21), (130), (221), (-202), and ($\overset{-}{1}$30). These characteristic peaks indicated that the prepared materials have better crystallinity. Moreover, the CNF was introduced into the CuWO_4_ materials the same peaks were observed. However, the characteristic peak position was shifted with decreasing the peak intensity. As per the XRD result, the CNF materials have successfully been incorporated with CuWO_4_. However, we cannot observe the CNF characteristic peaks due to the lower concentration of CNF. The average crystallite size of the CuWO_4_ was ~48.25 nm and the CuWO_4_/CNF was ~46.73 nm, respectively.

The Raman characterization was used to analyze the properties of CuWO_4_, CNF, and CuWO_4_/CNF. The Raman spectra of CuWO_4_ showcase intense peaks at 593, 770, and 900 cm^−1^ in Figure 1b. Hardcastle and Wachs’s rules state that there are six internal and external modes connected to WO_4_ octahedra in the composite [41]. The Raman shift at 900 cm^−1^ matches to W-O stretching vibration mode present in the anorthic structure of CuWO_4_ [39]. The peaks at 1374 cm^−1^ and 1609 cm^−1^ correspond to the D and G modes of CNF spectra. The amount of disorder in the carbon material can be determined by comparing the intensity of the D peak and the G peak. Because the metal on the surface of the carbon fiber disturbs the interior atomic ordering, the ID/IG value is 1.138 [42]. Therefore, the formation of CuWO_4_/CNF has been confirmed using Raman spectroscopy.

### 3.2. Structure and Morphology Analysis by FESEM and HRTEM Studies

**Figure 2:**
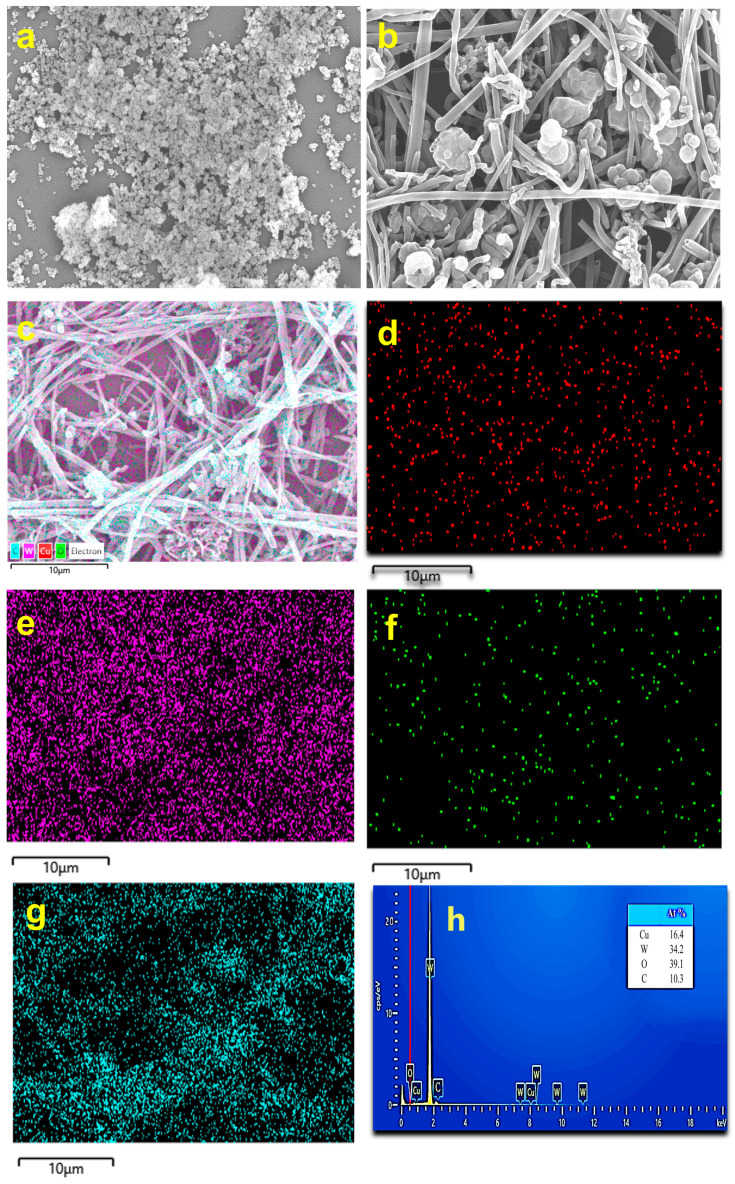
FESEM analysis for (**a**) CuWO_4_, (**b**) CuWO_4_/CNF, (**c**) EDX color mapping for CuWO_4_/CNF, and (**d**) Cu, (**e**) W, (**f**) O, and (**g**) C elemental mapping (**h**) EDX spectra of CuWO_4_/CNF composite.

Figure 2a–c shows the FESEM analysis was employed to examine the structural morphology of the synthesized CuWO_4_ and CuWO_4_/CNF composite. The CuWO_4_ particle has a spherical shape structure with uniform size within the nanometer range. Figure 2a–c clearly shows that the CuWO_4_/CNF composite has successfully formed, and CuWO_4_ materials have been placed on the CNF surface. In addition, the presence of all elements was examined by FESEM-EDX analysis with elemental mapping. The elemental mapping has revealed that all elements are uniformly distributed to the materials (Figure 2d–g). The EDX spectra of the CuWO_4_/CNF composite confirm the presence of Cu (16.4%), W (32.2%), O (39.1%), and C (10.3%) atom base in mass fraction of C and other elements, as shown in Figure 2h.

**Figure 3:**
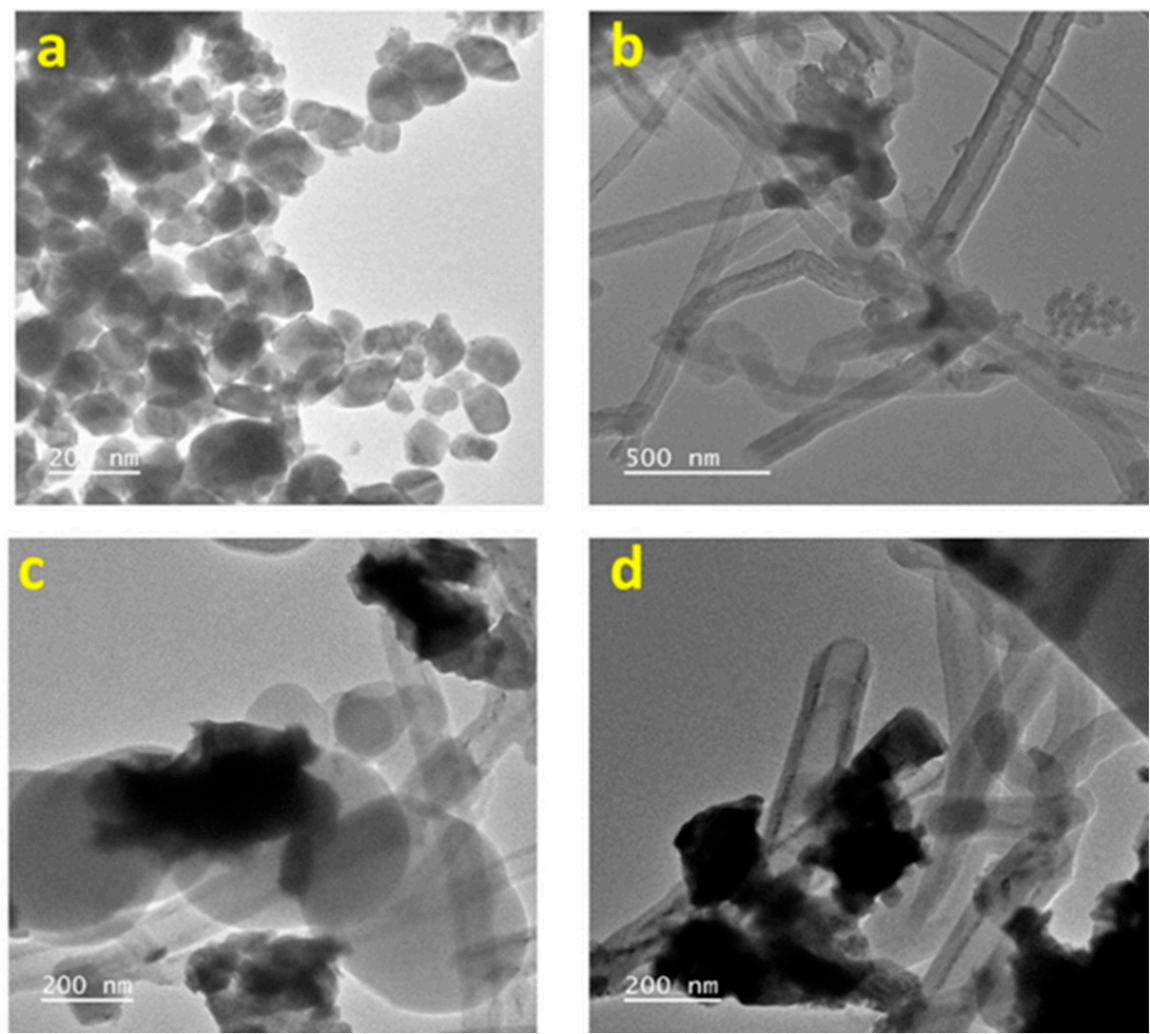
HR-TEM analysis of (**a**) CuWO_4_, (**b**) CNF, and (**c**,**d**) CuWO_4_/CNF nanocomposite.

Therefore, the synthesized materials have a uniform structure with the size of nano-range. The topology of synthesized nanocomposites was studied by HRTEM analysis, which is shown in Figure 3a–d. Figure 3a shows The HR-TEM analysis revealed that the CuWO_4_ materials have formed a potato-like structure with uniform size. The average particle size of the prepared CuWO_4_ has ~100 nm. Figure 3b shows the HRTEM image of CNF, which highlights that the CNF has a hollow-tube-like structure with a diameter of ~50 nm. In addition, Figure 3c,d shows that CuWO_4_ and CNF have successfully incorporated and formed the CNF/CuWO_4_ composite, and CuWO_4_ nanoparticles have been successfully placed on the surface of the CNF hollow tube.

## 4. Electrochemical Reduction Studies of 4-NT on the GCE/CNF/CuWO_4_ Electrode

### 4.1. Electrochemical Behavior of Different Modified Electrodes

**Figure 4:**
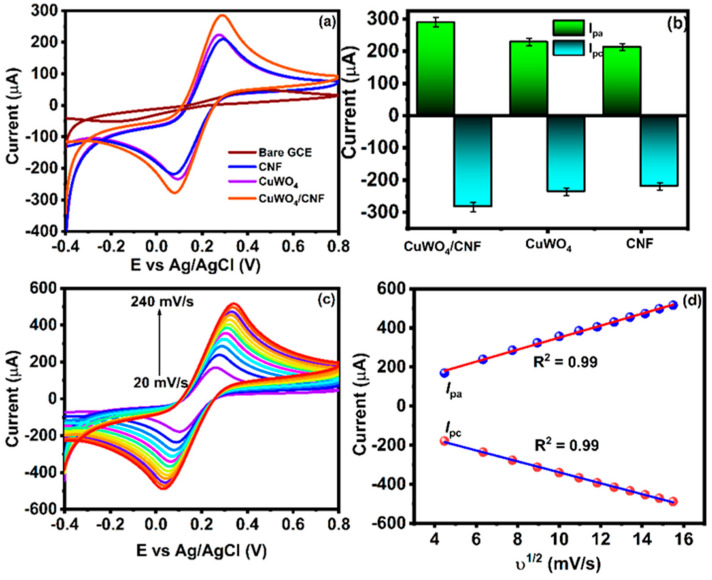
(**a**) Cyclic voltammogram of bare GCE, CNF/GCE, CuWO_4_/GCE, CuWO_4_/CNF/GCE modified electrodes in 5 mM K_3_[Fe (CN)_6_]^3−/4−^ 0.1 M KCl system at 10 mV/s. (vs. Ag/AgCl). (**b**) Bar diagram of redox currents for different electrodes. (**c**) CV profiles were recorded for CuWO_4_/CNF/GCE at scan rates of 20–240 mVs^−1^. Different colors peak mentioned different scan rates studies. (**d**) The linear plot for scan rate vs. peak current (*I*_p_).

The electrochemical properties of synthesized CuWO_4_/CNF/GCE electrodes were probed using the CV technique. Figure 4a presents the attained CV signals of bare GCE, CNF/GCE, CuWO_4_/GCE, and CuWO_4_/CNF/GCE at the sweep rate of 50 mV/s in 5.0 mM of [Fe(CN)6]^3−/4−^ constituting 0.1 KCl as an electrolyte solution. It can be seen that bare GCE displays a weak, reversible, redox peak which is negligible due to lower current density. Concurrently, CNF/GCE (*I*_pc_ = −290, *I*_pa_ = 284), CuWO_4_/GCE (*I*_pc_ = −229, *I*_pa_ = 237), CuWO_4_/CNF/GCE (*I*_pc_ = −213, *I*_pa_ = 220) contributes to the well-determined redox behavior with a higher current density. These outcomes reveal that there is elevated electrochemical behavior when the GCE is modified with CuWO_4_/CNF nanocomposite. Further, the electroactive surface area (EASA) of the fabricated electrodes was calculated using the Randles–Sevick equation. Figure 4b shows the bar diagram of redox currents for CNF/GCE, CuWO_4_/GCE, and CuWO_4_/CNF/GCE electrodes. Figure 4c shows the CV curves for different sweep rates from 20–240 mV/s for CuWO_4_/CNF/GCE. Figure 4d shows the linear relation of peak current versus the square root of the scan rate. On substitution of these values in the Randles–Sevick equation, the EASA of the CuWO_4_/CNF/GCE nanocomposite electrode was found to be 1.107 cm^2^, which is greater than other fabricated electrodes. Hence, the CuWO_4_/CNF/GCE electrodes hold a greater surface area with more active sites, which can promote the electrochemical interaction toward the detection of 4-NT.

### 4.2. Different Films and Effects of Different Concentration Studies 

**Figure 5:**
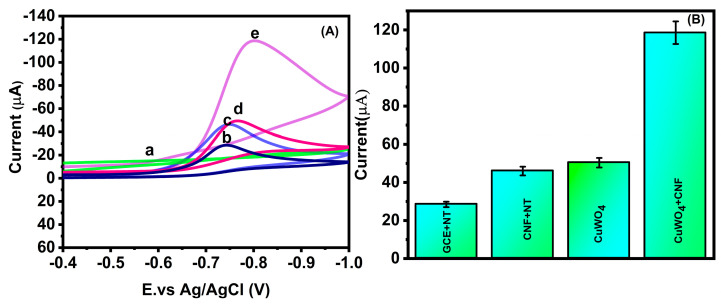
(a) Cyclic voltammetry detection of 4-NT using different electrodes (b) GCE, (c) CNF/GCE, (d) CuWO_4_/GCE, (e) CuWO_4_/CNF/GCE in 0.05 M PBS (pH = 7.0) with 100 µM 4-NT. (**A**) shows the CV response in the non-existence of 4-NT, (**B**) Bar diagram of different electrode peak currents.

**Scheme 1:**
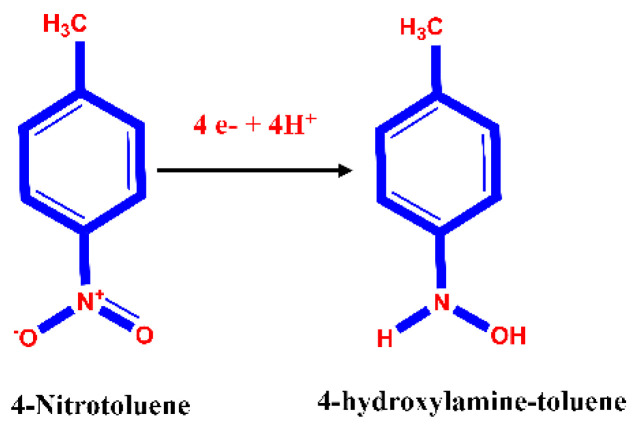
The electrocatalytic reduction of 4-nitro toluene to 4-hydroxylamine toluene on the CuWO_4_/CNF/GCE electrode surface.

The electrocatalytic response of CuWO_4_/CNF nanocomposite was evaluated using cyclic voltammetry to approach the detection of 4-NT. Figure 5A depicts the CV response of (b) bare GCE, (c) CNF/GCE, (d) CuWO_4_/GCE, (e) CuWO_4_/CNF/GCE nanocomposite modified electrode in the presence of 100 μM 4-NT, containing N_2_ saturated 0.05 M PBS at the fixed scan rate of 50 mV/s. (a) shows the CV response in the non-existence of 4-NT. Although, the CuWO_4_/CNF nanocomposite-modified electrode depicts a greater reduction-peak current towards the reduction of 4-NT, and at the same time, there is no prominent reduction-peak current perceived for the non-existence of 4-NT, while the bare GCE reduced at the lowest 4-NT reduction potential (*E*_pc_) and cathodic current (*I*_pc_) of −0.73 V and 29.07 μA, respectively. In addition, the CNF shows the negative shift of *E*_pc_ at −0.74 V and enhanced *I*_pc_ at about 45.65 μA, which accounts for the interaction of analyte molecules with a larger surface area. Furthermore, the *E*_PC_ and *I*_pc_ of CuWO_4_ were observed at −0.76 V and 49.73 μA, distinctively, which is attributed to the high-electrocatalytic property of the CuWO_4_ nanoparticles. Over and above that, there was an excellent upraised *E*_pc_ at −0.80 V and *I*_pc_ around 118. 83 μA (Figure 5B). The combined effect, such as good electrocatalytic activity of CuWO_4_ and the larger surface area with high-electron mobility of CNF, exhibited higher *I*_pc_. The irreversible cathodic peak occurs due to the addition of 4-NT, which is further reduced to N-p-tolyl hydroxylamine (4-hydroxylamine-toluene) (Scheme 1) [43].

**Figure 6:**
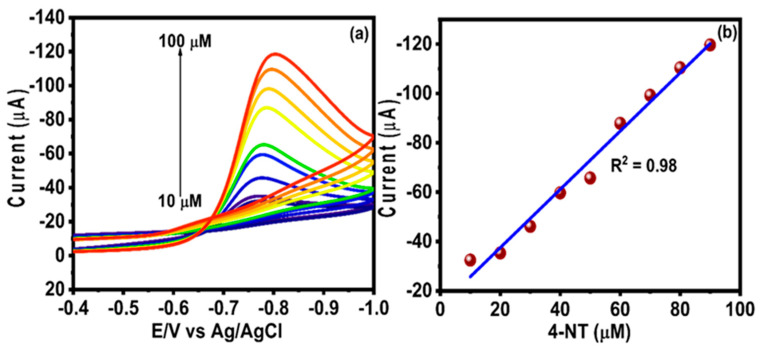
(**a**) Cyclic voltammetry response of CNF/CuWO_4_ at different concentrations of 4-NT from 10 μM to 100 μM in 0.05 M PBS (pH = 7.0) at the scan rate 50 mV/s. Different colors mentioned for different concentration. (**b**) Linear plot of 4-NT peak current vs. concentration.

To examine the sensing ability of the nanocomposite towards different concentrations of the 4-NT analyte, independent studies are executed. Figure 6a shows the apparent sharp cathodic peak of 4-NT as the concentration was raised from 10 μM to 100 μM; there was a linear responsive current. Figure 6b shows the linear plot of the reduction peak current vs. 4-NT concentration.

### 4.3. Effect of Different pH and Different Scan Rate Studies

**Figure 7:**
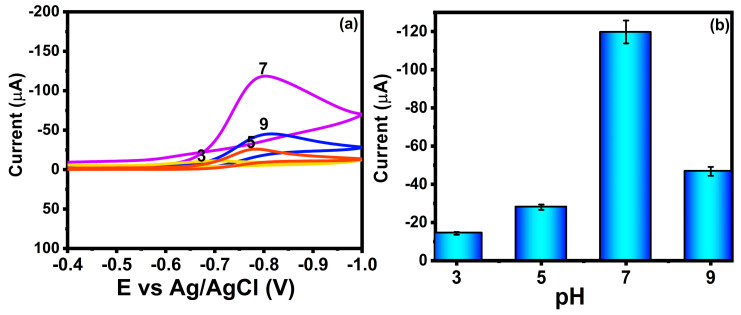
(**a**) Cyclic voltammetry response of CuWO_4_/CNF modified electrode in different pH-3, 5, 7, 9 conditions with 100 µM at the scan rate of 50 mV/s. (**b**) Calibration plot of reduction peak current vs. different pH.

The supporting electrolyte pH has a remarkable effect on the electrochemical reduction of 4-NT and was examined in the presence of 100 μM with different pH values from 3.0–7.0 of 0.05 M phosphate-buffered solution at the scan rate of 50 mV/s, as shown in Figure 7a. It can be seen that the cathodic peak of 4-NT gradually increases from pH 3.0 to 7.0 and attained the maximal cathodic peak current at pH 7.0. Moreover, there is a considerable decrease in peak current on increasing pH from 7.0 to 9.0. Therefore, a high current is found in pH 7.0, which is attributed to the Zwitterion effect [44]. Figure 7b shows the calibration curve of reduction peak current vs. different pH. Hence, pH 7.0 is chosen as the optimal pH for further investigation of electrochemical studies.

**Figure 8:**
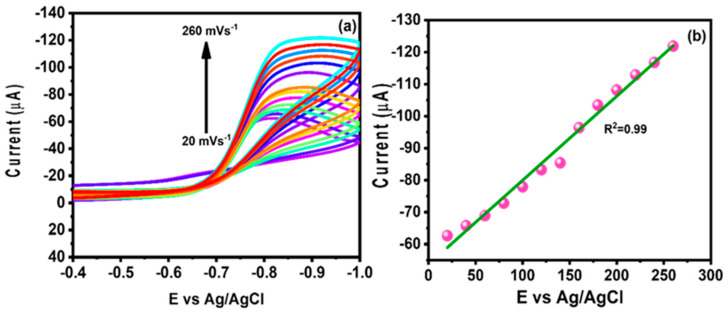
(**a**) Cyclic voltammetry response of CuWO_4_/CNF nanocomposite electrode in PBS with 100 µM of 4-NT at different scan rates. Different scan rates peaks mentioned in different colors. (**b**) Calibration plot of the reduction peak current vs. scan rate.

The effect of the scan rate was determined by the cyclic voltammetry technique with a CuWO_4_/CNF/GCE electrode in 0.05 M PBS, on the addition of 100 µM of 4-NT. Figure 8a displays that the cathodic peak current (*I*_pc_) gradually raised when the scan rate increased from 20 mV/s to 260 mV/s. It can be seen that by increasing the scan rate, there is a noticeable shift in negative direction. Figure 8b shows the calculation plot reduction peak current versus the scan rate. (IPC, R^2^ = 0.99). In low sweep rates, there is an easy interaction of the CuWO_4_/CNF/GCE electrode with the analyte 4-NT through electrostatic interaction. At the same time, for high-sweep rates, there is no sufficient time for electrostatic interaction between CuWO_4_/CNF/GCE electrode and the analyte 4-NT. The results show that the reduction of 4-NT is a surface-controlled process.

### 4.4. Differential Pulse Voltammetry Studies

**Figure 9:**
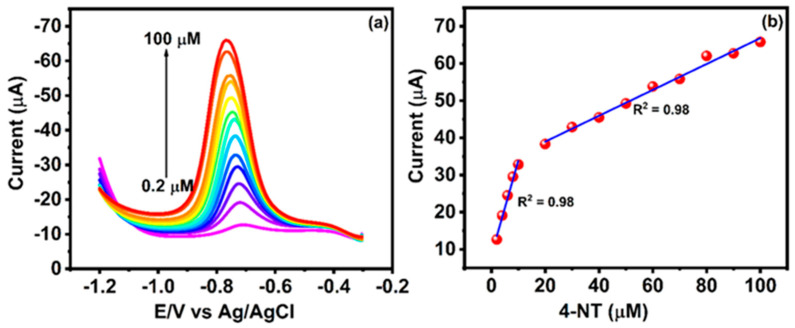
(**a**) DPV response of the CuWO_4_/CNF/GCE for consecutive addition of 4-NT in 0.05M PBS (pH = 7). Different concentration peaks mentioned in different colors. (**b**) The linear plot for cathodic current peak vs. concentration of 4-NT.

Differential pulse voltammetry is extremely sensitive for the effective quantitative determination of 4-NT. Accordingly, DPV is nominated for the determination of 4-NT. Figure 9a demonstrates the electrochemical examination of 4-NT from lower to higher concentrations (10 μM to 150 μM) in 0.05 M PBS. It can be seen that a sharp and apparent peak was observed on a lower to higher concentration increase. Figure 9b shows a linear relation between the 4-NT concentration and the cathodic peak current. In addition, the limit of detection (LOD) and sensitivity was calculated from the slope of the cathodic current. The CuWO_4_/CNF/GCE electrode exhibited the value of LOD and sensitivity was calculated to be 86.15 nM and 1.1485 μAμM^−1^cm^−2^, respectively. Finally, the CuWO_4_/CNF/GCE electrode performance was compared with the previously reported 4-NT sensor and mentioned in Table 1. As per Table 1, Mohammad. A. et al. used the ZnO-CN/GCE electrode, which exhibited the LOD of 100 nm by employing the CV technique [43]. However, Rani. S. et al. reported using AgNPs/Au electrodes with a linear range of 0.010–0.100 and LOD of 92 nm, using an amperometric technique [45]. Moreover, Yuan. S et al. used an NH_2_-Fe-MIL-88B@OMC-3 electrode and displayed the linear range of 20.00–225.0 with the LOD 8000 nm under the DPV technique [46]. Furthermore, Ahmad. K. et al. fabricated a GCE electrode with α-MnO_2_ that reached the linear range of 0.162–48.80 with an LOD of 144.0 using the CV technique [47]. Yang. R et al. obtained the linear range of 0.5–4, 0.2–1.2 and LOD of 442, 282.2 using the Pn-MWCNTs/Po-MWCNTs electrode [48]. On comparing the aforementioned studies, this CuWO_4_/CNF/GCE electrode shows a wide linear range of 0.2–100 with better sensitivity and LOD of 86.16 nM. Table 1 showcases a comparison of various parameters of previous works toward the detection of 4-NT by the CuWO_4_/CNF/GCE electrode.

### 4.5. Interference Studies

**Figure 10:**
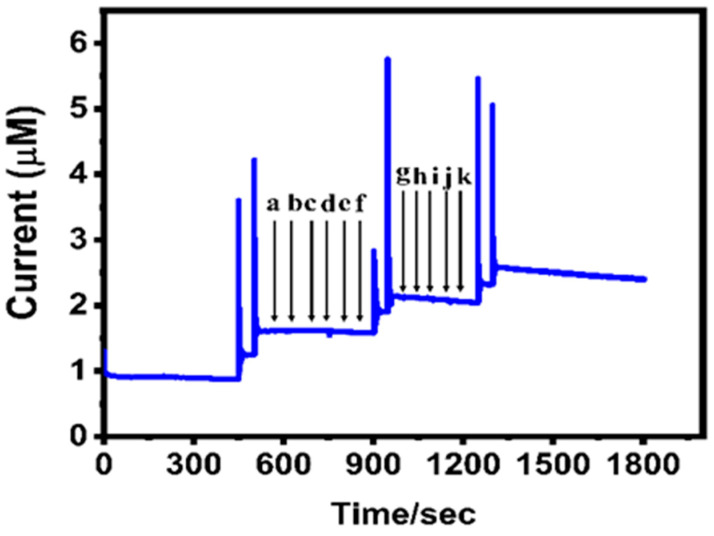
i–t response of reduction of 4-NT in the presence of various interfering ions anions such as (a) F^−^, (b) Cl^−^, (c) NO_3_^−^, (d) CO_2_^−^, (e) CO_3_^−^, (f) Cl^−^, and cations such as (g) Na^+^, (h) Cr^2+^, (i) Mg^2+^, (j) Zn^+^, (k) NH_4_^+^.

For a newly constructed sensor, selectivity is a key component. The selectivity of the as-prepared CuWO_4_/CNF/GCE-modified electrode was examined in the presence of anions such as (a) F^−^, (b) Cl^−^, (c) NO_3_^−^, (d) CO_2_^−^, (e) CO_3_^−^, (f) Cl^−^, and cations such as (g) Na^+^, (h) Cr^2+^, (i) Mg^2+^, (j) Zn^+^, (k) NH_4_^+^, using the amperometric technique. Figure 10 shows that even after the 25-fold excess concentration of other interfering ions than 4-NT, there was a sharp prominent response only for the reduction of 4-NT; there is no response for the addition of other aforementioned interfering ions. Hence, the above outcomes indicate that the as-synthesized CuWO_4_/CNF/GCE electrode can be used for the selective detection of 4-NT even in the presence of other ions.

### 4.6. Repeatability, Reproducibility, and Stability Performance of GCE/CuWO_4_/CNF Electrode

**Figure 11:**
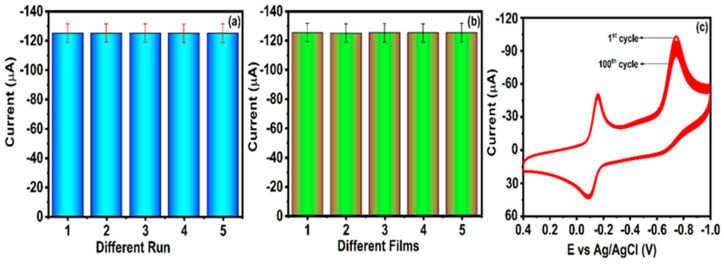
(**a**) Repetitive runs of a single GCE/CuWO_4_/CNF electrode. (**b**) Reproducibility of CuWO_4_/CNF/GCE electrode for five differently fabricated electrodes. (**c**) Cyclic stability of the CuWO_4_/CNF/GCE electrode in 0.05 M PBS with 100 µM at the scan rate of 50 mV/s for 100 cycles.

The repeatability, reproducibility, and stability of CuWO_4_/CNF/GCE-modified electrodes toward the detection of 4-NT were studied. Figure 11a shows that the repeatability was examined by the addition of 100 µM of 4-NT in N_2_ saturated 0.05 M PBS solution at the scan rate of 50 mV/s for five successive runs. Further, Figure 11b exhibits the reproducibility studied for five different electrodes in N_2_ saturated 0.05 M PBS solution at the scan rate of 50 mV/s in the presence of 100 µM. In addition, Figure 11c shows the stability of the as-synthesized CuWO_4_/CNF nanocomposite, and was investigated for 100 cycles. The results showed that there was less than 10% current loss. From these stability studies, we can conclude that the CuWO_4_/CNF/GCE-modified electrode shows better repeatability, reproducibility, and stability results.

### 4.7. Real Sample Analysis

**Figure 12:**
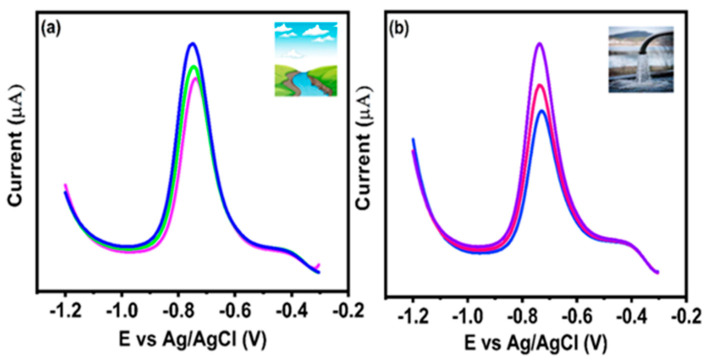
DPV voltammogram for various concentrations from 10 to 30 µM in water samples of (**a**) river water and (**b**) tap water.

To investigate the practical sensibility and applicability of the samples towards the detection of 4-NT, real sample analysis was performed, which is demonstrated in Figure 12a,b, and the results are mentioned in Table 2. Before executing the real sample analysis, water samples were collected from rivers and taps. The collected water samples are centrifuged and then diluted with PBS to circumvent any interference that is present in the river and tap water samples. Then, the calculated amount of 4-NT was spiked into the solution and real sample analysis was performed using DPV. Furthermore, using the standard addition method, the recovery percentage (91.51–97.10%) was determined with an RDS value of less than 10%. Thus, the prepared CuWO_4_/CNF/GCE electrode can be a purposeful electroactive material for the detection of 4-NT in water samples with a good recovery percentage.

## 5. Conclusions

In this study, CuWO_4_ was synthesized using the hydrothermal method. Furthermore, the CuWO_4_ particles were incorporated into CNF using the ultra-sonication method. To confirm the formation, crystallinity, phase purity, structure morphology, and topology of the as-synthesized nanoparticles, XRD, FESEM, EDS, and HR-TEM analyses were performed. The electrochemical ability was conducted using cyclic voltammetry and differential pulse voltammetry studies. The GCE/CNF/CuWO_4_ electrode possessed an appreciable sensibility towards the detection of 4-NT with a better LOD of 86.16 nM with a sensitivity of 7.258 μA μM^−1^ cm^−2^, also, a wide linear range of 0.2–100 µM was observed. To add on, the developed sensor exhibited impressive repeatability, reproducibility, and stability for 4-NT detection. We also achieved good recovery results in real-time analysis of 4-NT. Hence, it can be applied as a potential electrochemical sensor for the detection of 4-NT in water samples.

## Figures and Tables

**Table 1:** Comparison of electrochemical detection 4-NT by of GCE/CuWO_4_/CNF electrode and various modified electrodes.

Electrodes	Techniques	Linear Range	LOD (nM)	Reference
ZnO-CN/GCE	CV	-	100.0	[43]
Ag NPs/Au	Amperometry	0.010–0.100	92.00	[45]
NH_2_-Fe-MIL-88B@OMC-3	DPV	20.00–225.0	8000	[46]
GCE/α-MnO_2_	CV	0.162–48.80	144.0	[47]
Pn-MWCNTs/Po-MWCNTs	SWASV	0.5–4, 0.2–1.2	442, 282.2	[48]
CuWO_4_/CNF/GCE	DPV	0.2–100	86.15	This work

**Table 2:** It shows the calculation of the recovery percentage and RSD results of the collected real samples: river and tap water samples.

Samples	Added (µM)	Found (µM)	Recovery (%)	RSD (%)
River water	10	9.15	91.51	0.121
	20	19.08	95.42	0.102
	30	29.08	96.95	0.070
Tap Water	10	9.18	91.80	0.079
	20	19.27	96.35	0.108
	30	29.13	97.10	0.089

## Data Availability

Data is available on request due to privacy/ethical restrictions.

## References

[1] Gou Z., Zhang X., Zuo Y., Tian M., Dong B., Lin W. (2019). Pyrenyl-Functionalized Polysiloxane Based on Synergistic Effect for Highly Selective and Highly Sensitive Detection of 4-Nitrotoluene. ACS Appl. Mater. Interfaces.

[2] Chakraborty U., Garg P., Bhanjana G., Kaur G., Kaushik A., Chaudhary G.R. (2022). Spherical Silver Oxide Nanoparticles for Fabrication of Electrochemical Sensor for Efficient 4-Nitrotoluene Detection and Assessment of their Antimicrobial Activity. Sci. Total Environ..

[3] Chakraborty U., Kaur I., Chauhan A., Kaur N., Kaur G., Chaudhary G.R. (2023). Zinc oxide-Copper Sulfide Semiconductor Nano-heterostructure for Low-Level Electrochemical Detection of 4-Nitrotoluene. Electrochim. Acta.

[4] Easwaramoorthy D., Yu Y.C., Huang H.J. (2001). Chemiluminescence Detection of Paracetamol by a Luminol-Permanganate Based Reaction. Anal. Chim. Acta.

[5] Sazinas R., Andersen S.Z., Li K., Saccoccio M., Krempl K., Pedersen J.B., Chorkendorff I. (2021). Towards Understanding of Electrolyte Degradation in Lithium-Mediated Non-aqueous Electrochemical Ammonia Synthesis with Gas chromatography-Mass Spectrometry. RSC Adv..

[6] Liu Y., Mills R.C., Boncella J.M., Schanze K.S. (2001). Fluorescent Polyacetylene Thin Film Sensor for Nitroaromatics. Langmuir.

[7] Selvan P.S., Gopinath R., Saravanan V.S., Gopal N., Kumar A.S., Periyasamy K. (2007). Simultaneous Estimation of Paracetamol and Aceclofenac in Combined Dosage Forms by RP-HPLC Method. Asian J. Chem..

[8] Hira S.A., Nallal M., Park K.H. (2019). Fabrication of PdAg Nanoparticles Infused Metal-Organic Framework for Electrochemical and Solution-Chemical Reduction and Detection of Toxic 4-Nitrophenol. Sens. Actuators B Chem..

[9] Liu Y., Zhu L., Zhang Y., Tang H. (2012). Electrochemical Sensoring of 2,4-Dinitrophenol by Using Composites of Graphene oxide with Surface Molecular Imprinted Polymer. Sens. Actuators B Chem..

[10] Chakraborty U., Bhanjana G., Adam J., Mishra Y.K., Kaur G., Chaudhary G.R., Kaushik A. (2020). A Flower-like ZnO–Ag_2_O Nanocomposite for Label and Mediator Free Direct Sensing of Dinitrotoluene. RSC Adv..

[11] Lim H.J., Chua B., Son A. (2017). Detection of Bisphenol a Using Palm-size Nano Aptamer Analyzer. Biosens. Bioelectron..

[12] Sangamithirai D., Ramanathan S. (2022). Electrochemical Sensing Platform for the Detection of Nitroaromatics Using g-C_3_N_4_/V_2_O_5_ Nanocomposites Modified Glassy Carbon Electrode. Electrochim. Acta.

[13] Alam M.W., Al Q.H.S., Souayeh B., Ahmed W., Albalawi H., Farhan M., Naeem S. (2022). Novel Copper-Zinc-Manganese Ternary Metal Oxide Nanocomposite as Heterogeneous Catalyst for Glucose Sensor and Antibacterial Activity. Antioxidants.

[14] Tian C.M., Jiang M., Tang D., Qiao L., Xiao H.Y., Oropeza F.E., Zhang K.H.L. (2019). Elucidating the Electronic structure of CuWO_4_ thin films for enhanced photoelectrochemical water splitting. J. Mater. Chem. A.

[15] Wang C., Fu M., Cao J., Wu X., Hu X., Dong F. (2020). BaWO_4_/g-C_3_N_4_ heterostructure with excellent bifunctional photocatalytic performance. J. Chem. Eng..

[16] Sriram B., Shajahan S., Hsu Y.F., Wang S.F., Haija M.A. (2023). Electrochemical Behavior of a Copper Tungstate-Fabricated Disposable Strip for the Rapid and Real-Time Detection. ACS Appl. Nano Mater..

[17] Ahmed J., Ahamad T., Ubaidullah M., Al-Enizi A.M., Alhabarah A.N., Alhokbany N., Alshehri S.M. (2019). RGO Supported NiWO_4_ Nanocomposites for Hydrogen Evolution Reactions. Mater. Lett..

[18] Xu X., Pei L., Yang Y., Shen J., Ye M. (2016). Facile Synthesis of NiWO_4_/Reduced Graphene Oxide Nanocomposite with Excellent Capacitive Performance for Supercapacitors. J. Alloys Compd..

[19] Mikhailik V.B., Kraus H., Miller G., Mykhaylyk M.S., Wahl D. (2005). Luminescence of CaWO_4_, CaMoO_4_, and ZnWO_4_ Scintillating Crystals Under Different Excitations. J. Appl. Phys..

[20] Habibi M.M., Mousavi M., Shadman Z., Ghasemi J.B. (2022). Preparation of a Nonenzymatic Electrochemical Sensor Based on a g-C_3_N_4_/MWO_4_ (M: Cu, Mn, Co, Ni) Composite for the Determination of H_2_O_2_. New J. Chem..

[21] Gaillard N., Chang Y., DeAngelis A., Higgins S., Braun A. (2013). A Nanocomposite Photoelectrode Made of 2.2 eV Band gap Copper Tungstate (CuWO_4_) and Multi-wall carbon nanotubes for Solar-Assisted Water Splitting. Int. J. Hydrogen Energy.

[22] Davi M., Mann M., Ma Z., Schrader F., Drichel A., Budnyk S., Slabon A. (2018). An MnNCN-Derived Electrocatalyst for CuWO_4_ Photoanodes. Langmuir.

[23] Shad N.A., Bajwa S.Z., Amin N., Taj A., Hameed S., Khan Y., Dai Z., Cao C., Khan W.S. (2019). Solution Growth of 1D Zinc Tungstate (ZnWO_4_) Nanowires; Design, Morphology, and Electrochemical Sensor Fabrication for Selective Detection of Chloramphenicol. J. Hazard. Mater..

[24] Dinh P.H., Pham T.D., Thuan D.V., Cam N.T.D., Hanh N.T., Van Ha H., Tri N.L.M. (2020). CuWO_4_ Decorated by Polypyrrole (PPy) Protector/Sensitizer for Novel Photocatalytic and Stable Water Splitting for Hydrogen Generation. Int. J. Hydrogen Energy.

[25] Hosseinpour M.S.M., Sobhani N.A. (2016). Simple Synthesis and Characterization of Copper Tungstate Nanoparticles: Investigation of Surfactant Effect and Its Photocatalyst Application. J. Mater. Sci. Mater. Electron..

[26] Kloprogge J.T., Weier M.L., Duong L.V., Frost R.L. (2004). Microwave-Assisted Synthesis and Characterisation of Divalent Metal Tungstate Nanocrystalline Minerals: Ferberite, Hubnerite, Sanmartinite, Scheelite and Stolzite. Mater. Chem. Phys..

[27] Yu S.-H., Liu B., Mo M.-S., Huang J.-H., Liu X.-M., Qian Y.-T. (2003). General Synthesis of Single-Crystal Tungstate Nanorods/Nanowires: A Facile, Low-Temperature Solution Approach. Adv. Funct. Mater..

[28] Bagwade P.P., Malavekar D.B., Ubale S.B., Bulakhe R.N., In I., Patil U.M., Lokhande C.D. (2022). Synthesis, Characterization and Supercapacitive Application of Nanocauliflower-like Cobalt Tungstate Thin Films by Successive Ionic Layer Adsorption and Reaction (SILAR) Method. Electrochim. Acta.

[29] Akhavan O., Ghaderi E., Rahighi R. (2012). Toward Single-DNA Electrochemical Biosensing by Graphene Nanowalls. ACS Nano.

[30] Bounegru A.V., Apetrei C. (2020). Carbonaceous Nanomaterials Employed in the Development of Electrochemical Sensors Based on Screen-Printing Technique-A Review. Catalysts.

[31] Yuan Y., Zhang F., Wang H., Gao L., Wang Z. (2018). A Sensor Based on Au Nanoparticles/Carbon Nitride/Graphene Composites for the Detection of Chloramphenicol and Ciprofloxacin. ECS J. Solid State Sci. Technol..

[32] Wang Z., Wu S., Wang J., Yu A., Wei G. (2019). Carbon Nanofiber-Based Functional Nanomaterials for Sensor Applications. J. Nanomater..

[33] Yang G., Zhao F. (2015). Electrochemical Sensor for Chloramphenicol Based on Novel Multiwalled Carbon Nanotubes@ Molecularly Imprinted Polymer. Biosens. Bioelectron..

[34] Huan K., Li Y., Deng D., Wang H., Wang D., Li M., Luo L. (2022). Composite-controlled electrospinning of CuSn bimetallic nanoparticles/carbon nanofibers for electrochemical glucose sensor. Appl. Surf. Sci..

[35] Gorska A., Zambrzycki M., Bator B.P., Piech R. (2022). New Electrochemical Sensor Based on Electrospun Carbon Nanofibers Modified with Cobalt Nanoparticles and Its Application for Atorvastatin Determination. J. Electrochem. Soc..

[36] Sakthinathan S., Rajakumaran R., Keyan A.K., Yu C.L., Wu C.F., Vinothini S., Chen S.M., Chiu T.W. (2021). Novel Construction of Carbon Nanofiber/CuCrO_2_ Composite for Selective Determination of 4-Nitrophenol in Environmental Samples and for Supercapacitor Application. RSC Adv..

[37] Anupriya J., Rajakumaran R., Chen S.M., Karthik R., Kumar J.V., Shim J.J., Lee J.W. (2022). Raspberry-Like CuWO_4_ Hollow Spheres Anchored on Sulfur-Doped g-C_3_N_4_ Composite: An Efficient Electrocatalyst for Selective Electrochemical Detection of Antibiotic Drug Nitrofurazone. Chemosphere.

[38] Pavitra V., Praveen B.M., Nagaraju G. (2023). Exploring the Spondias Mombin (Hog plum) Mediated ZnWO_4_–CuWO_4_ Nanocomposite for Photocatalysis and Electrochemical Nitrite Sensing. Mater. Chem. Phys..

[39] Karthika A., Karuppasamy P., Selvarajan S., Suganthi A., Rajarajan M. (2019). Electrochemical Sensing of Nicotine Using CuWO_4_ Decorated Reduced Graphene oxide Immobilized Glassy Carbon Electrode. Ultrason. Sonochem..

[40] Nong C., Yang B., Li X., Feng S., Cui H. (2022). An Ultrasensitive Electrochemical Immunosensor Based on In-situ Growth of CuWO_4_ Nanoparticles on MoS_2_ and Chitosan-Gold Nanoparticles for Cortisol Detection. Microchem. J..

[41] Ruiz-Fuertes J., Errandonea D., Segura A., Manjón F.J., Zhu Z., Tu C.Y. (2008). Growth, Characterization, and High-Pressure Optical Studies of CuWO_4_. High Press. Res..

[42] Lin J., Mei Q., Duan Y., Yu C., Ding Y., Li L. (2020). A Highly Sensitive Electrochemical Sensor based on Nanoflower-like MoS_2_-Ag-CNF Nanocomposites for the Detection of VB2. J. Nanopart. Res..

[43] Mohammad A., Ahmad K., Qureshi A., Tauqeer M., Mobin S.M. (2018). Zinc Oxide-Graphitic Carbon Nitride Nanohybrid as an Efficient Electrochemical Sensor and Photocatalyst. Sens. Actuators B Chem..

[44] Xiao Q., Lu S., Huang C., Su W., Zhou S., Sheng J., Huang S. (2017). An Electrochemical Chiral Sensor Based on Amino-Functionalized Graphene Quantum Dots/β-Cyclodextrin Modified Glassy Carbon Electrode for Enantioselective Detection of Tryptophan Isomers. J. Iran. Chem. Soc..

[45] Rani S., Dilbaghi N., Kumar S., Varma R.S., Malhotra R. (2020). Rapid Redox Sensing of P-Nitrotoluene in Real Water Samples Using Silver Nanoparticles. Inorg. Chem. Commun..

[46] Yuan S., Bo X., Guo L. (2018). In-Situ Growth of Iron-Based Metal-Organic Framework Crystal on Ordered Mesoporous Carbon for Efficient Electrocatalysis of p-Nitrotoluene and Hydrazine. Anal. Chim. Acta.

[47] Ahmad K., Mohammad A., Mobin S.M. (2017). Hydrothermally Grown α-MnO_2_ Nanorods as Highly Efficient Low-Cost Counter-Electrode Material for Dye-Sensitized Solar Cells and Electrochemical Sensing Applications. Electrochim. Acta.

[48] Yang R., Wei Y., Yu Y., Gao C., Wang L., Liu J.H., Huang X.J. (2012). Make It Different: The Plasma Treated Multi-Walled Carbon Nanotubes Improve Electrochemical Performances toward Nitroaromatic Compounds. Electrochim. Acta.

